# Supplementary material for: Structural insight into the intraflagellar transport complex IFT-A and its assembly in the anterograde IFT train
Source: Nat Commun. 2023 Mar 17;14:1506. doi: 10.1038/s41467-023-37208-2 (PMC10023715; doi:10.1038/s41467-023-37208-2)
Supplement: Supplementary file 1 — Supplementary Information [file 41467_2023_37208_MOESM1_ESM.pdf]

**Supplementary Information**

# Structural insight into the intraflagellar transport complex IFT-A and its assembly in the anterograde IFT train

Yuanyuan Ma[1,2#], Jun He[1,2#], Shaobai Li[1,2], Deqiang Yao[3], Chenhui Huang[1,2], Jian Wu[1,2*] and Ming Lei[1,2,4*]

[1]Ninth People's Hospital, Shanghai Jiao Tong University School of Medicine, Shanghai 200011, China.

[2]Shanghai Institute of Precision Medicine, Shanghai 200125, China.

[3]Renji Hospital, Shanghai Jiao Tong University School of Medicine, Shanghai 200032, China.

[4]State Key Laboratory of Oncogenes and Related Genes, Shanghai Jiao Tong University School of Medicine, Shanghai 200025, China.

[#]These authors contributed equally to this work.

*Correspondence: M.L. (leim@shsmu.edu.cn) and J.W. (wujian@shsmu.edu.cn)

## Supplementary Fig. 1

**a**

| Protein name | Uniprot Accession Number | MW(kDa) | Peptide spectrum matches | Unique peptides |
|---|---|---|---|---|
| IFT121 | Q22U89 | 137.8 | 145 | 74 |
| IFT139 | I7MFN3 | 153.9 | 116 | 67 |
| IFT144 | Q22BP2 | 158.1 | 114 | 65 |
| IFT140 | I7LVZ7 | 161.4 | 101 | 55 |
| IFT122 | Q244W3 | 143.7 | 86 | 55 |
| IFT43 | Q22NF5 | 16.7 | 3 | 3 |

**b**

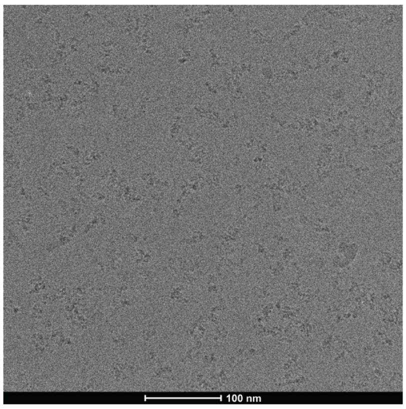

**Supplementary Fig. 1 | Biochemical and cryo-EM analyses of the purified IFT-A complex.** **a,** Mass spectrometry analysis of the purified IFT-A complex. **b,** A representative cryo-EM image of IFT-A. Each experiment was repeated three times independently with similar results. Source data are provided as a Source Data file.

**Supplementary Fig. 2**

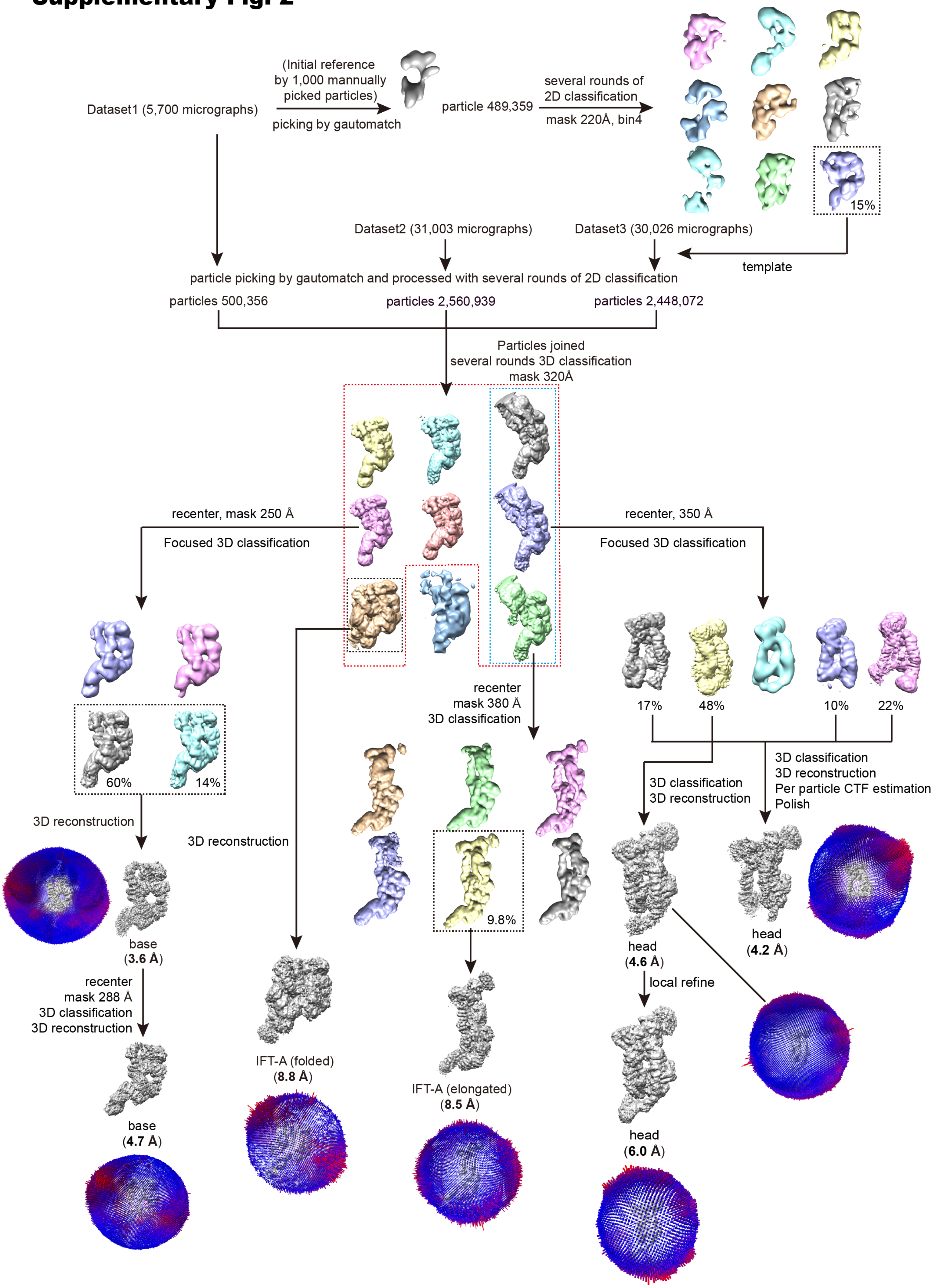

**Supplementary Fig. 2 | Structure determination of the IFT-A complex by cryo-EM.**

Cryo-Image processing flowchart, reconstruction of the IFT-A complex and angular distribution of reconstructed particles.

# Supplementary Fig. 3

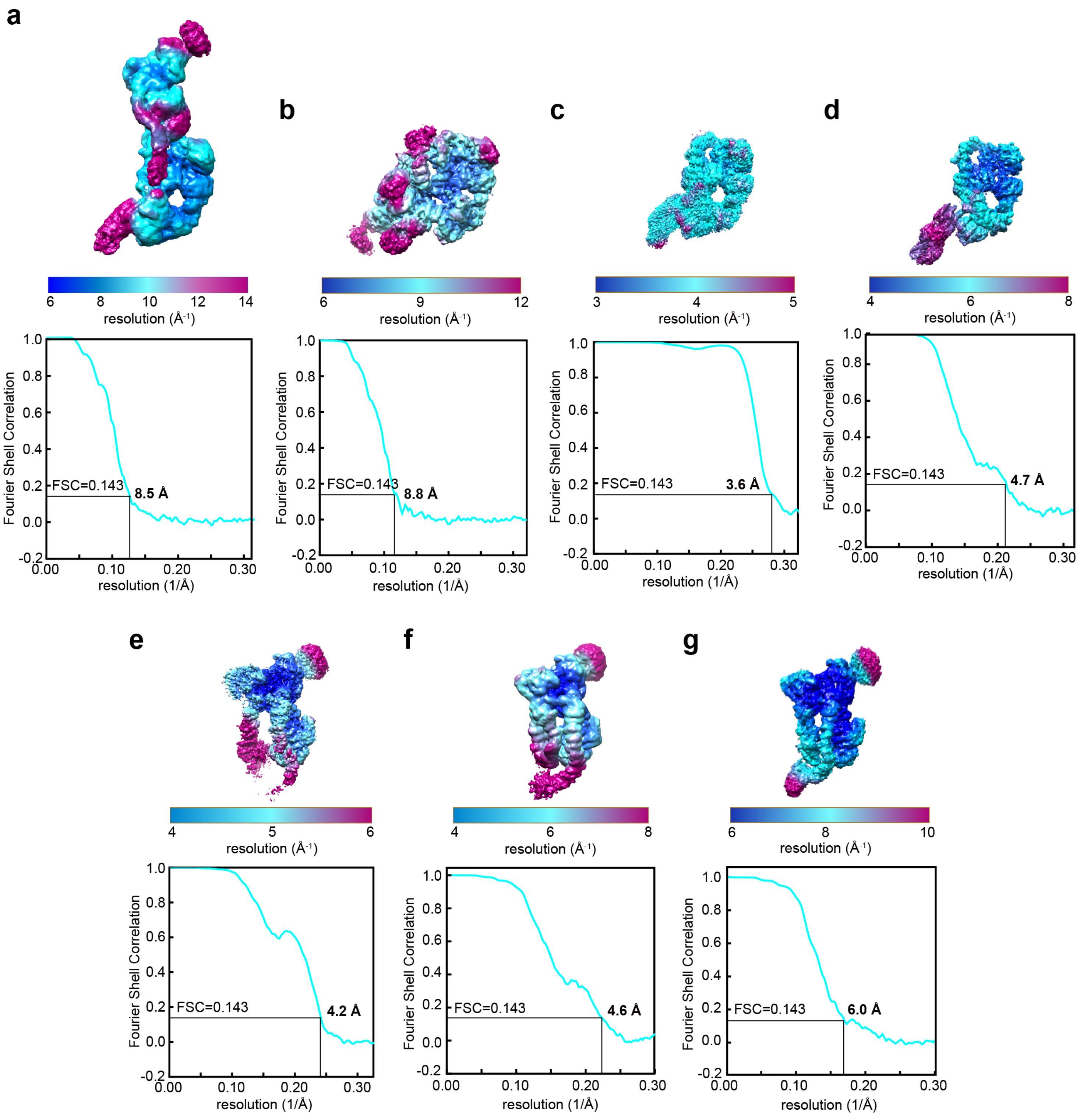

**Supplementary Fig. 3 | Local resolution estimation of IFT-A. a-g,** Local resolution maps and the corresponding Fourier Shell Correlation curves for IFT-A in elongated state (a), IFT-A in folded state (b), base module at 3.6 Å (c), base module at 4.7 Å (d), head module at 4.2 Å (e), head module at 4.6 Å (f) and head module at 6.0 Å (g).

# Supplementary Fig. 4

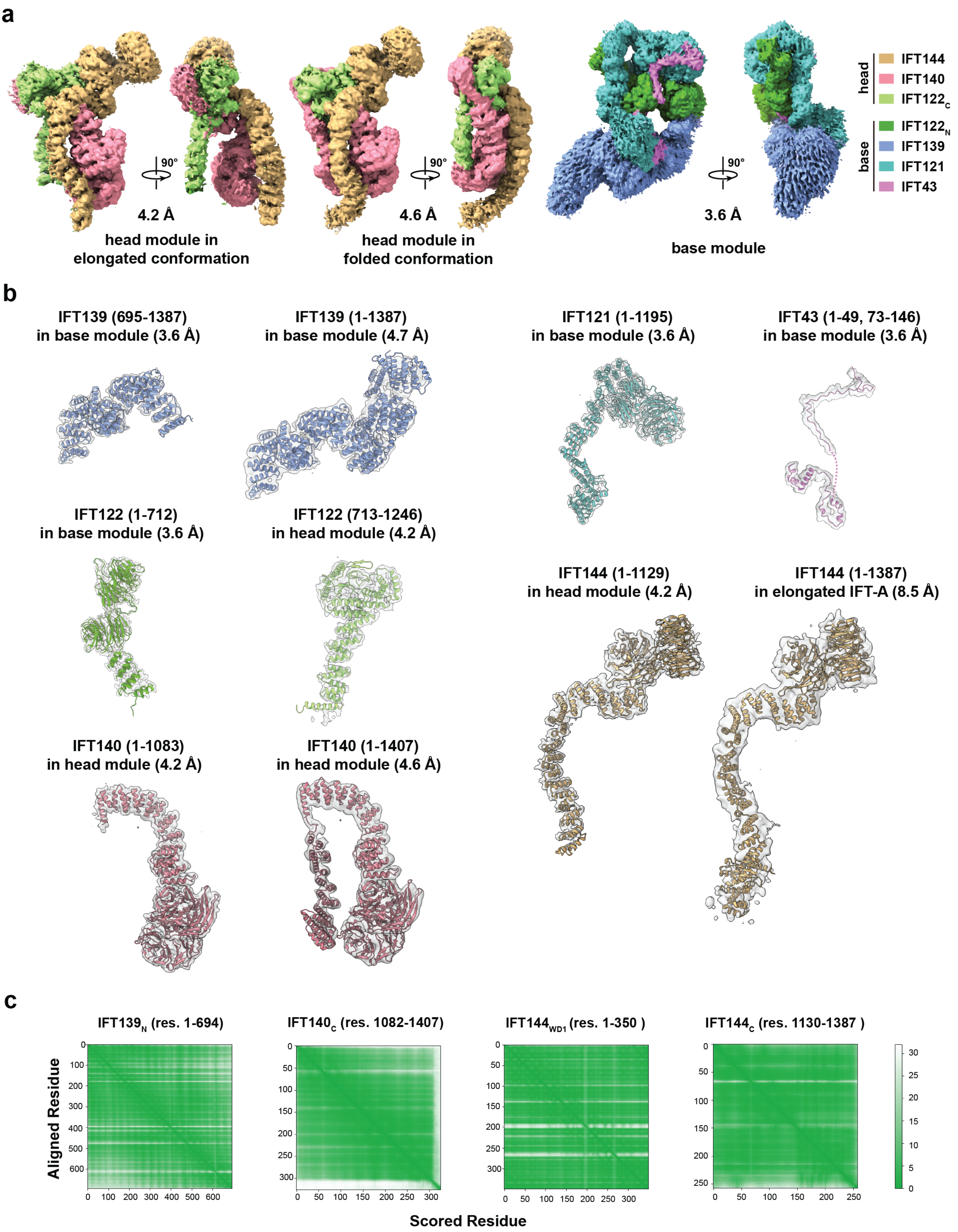

**Supplementary Fig. 4 | Cryo-EM density maps of the IFT-A complex. a,** High-resolution density maps of the head and base modules in two orthogonal views. The IFT-A components are colored and the color scheme is shown on the right. **b,** Cryo-EM density maps of the IFT-A subunits with the atomic models in cartoon representation. **c,** Predicted aligned error (PAE) plots of AlphaFold-2 predicted IFT139$_{N}$, IFT140$_{C}$, IFT144$_{WD1}$ and IFT144$_{C}$.

# Supplementary Fig. 5

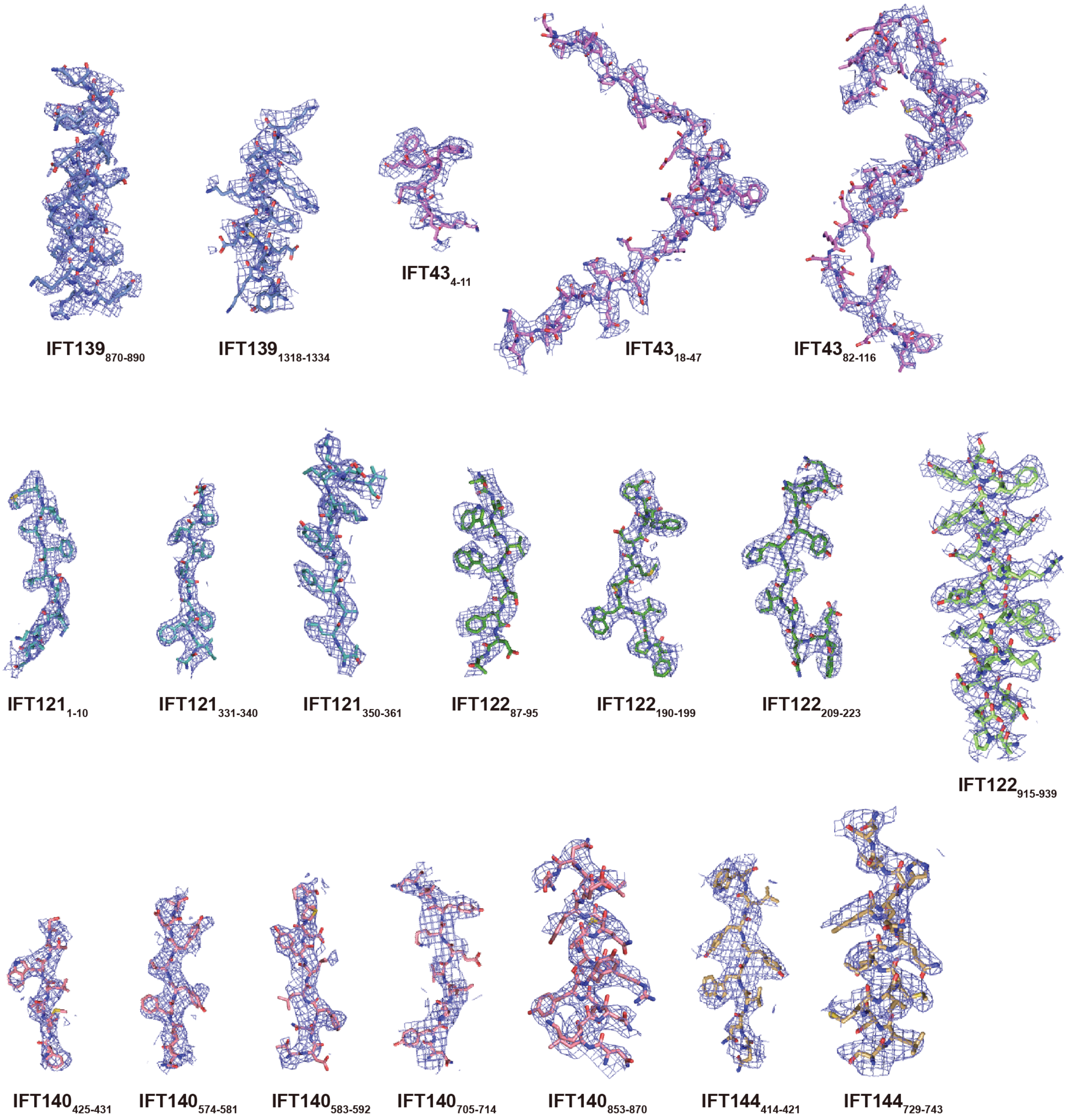

**Supplementary Fig. 5 | Representative cryo-EM density maps for the selected elements of each component of IFT-A.**

**Supplementary Fig. 6**

a

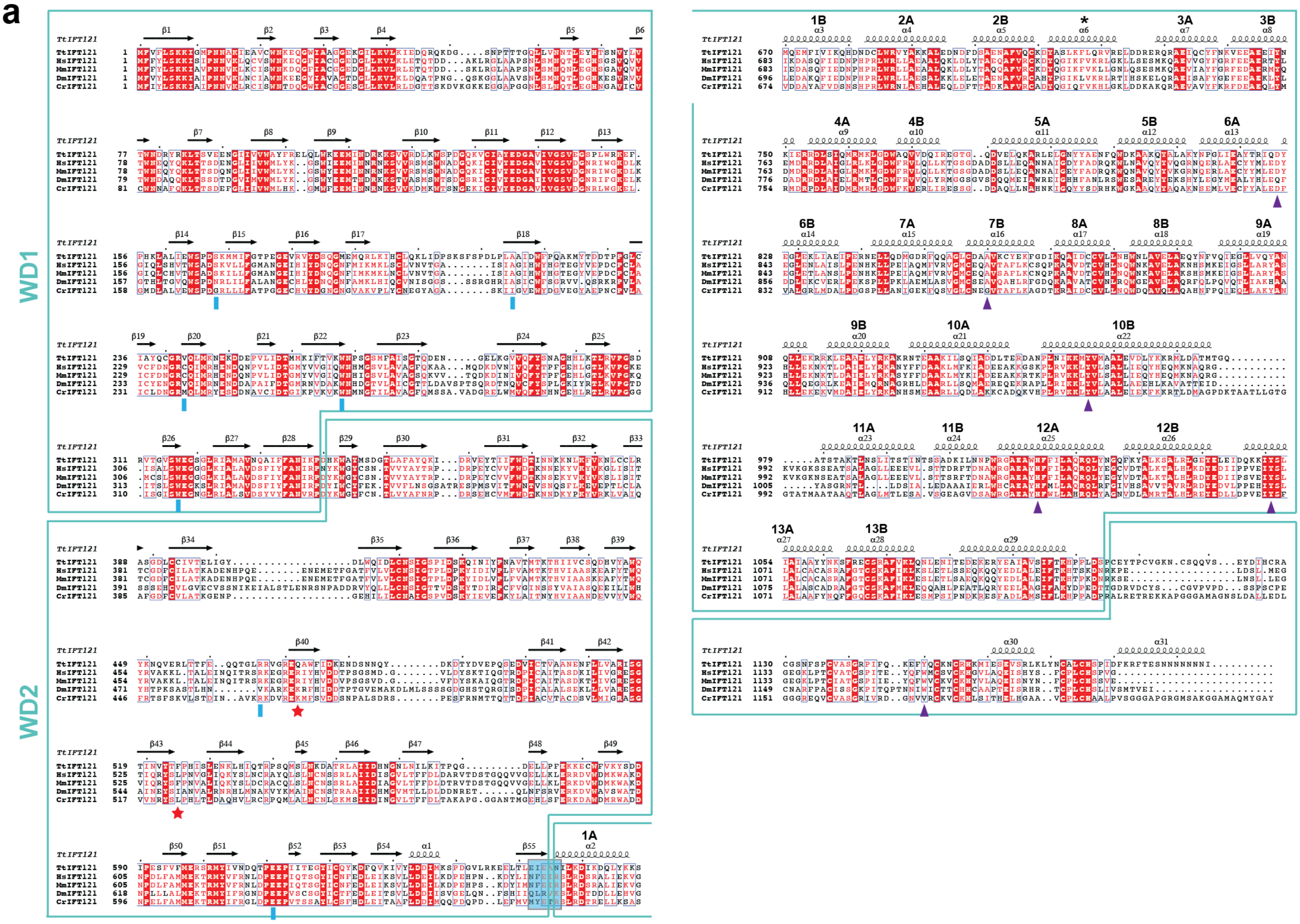


IFT121

b

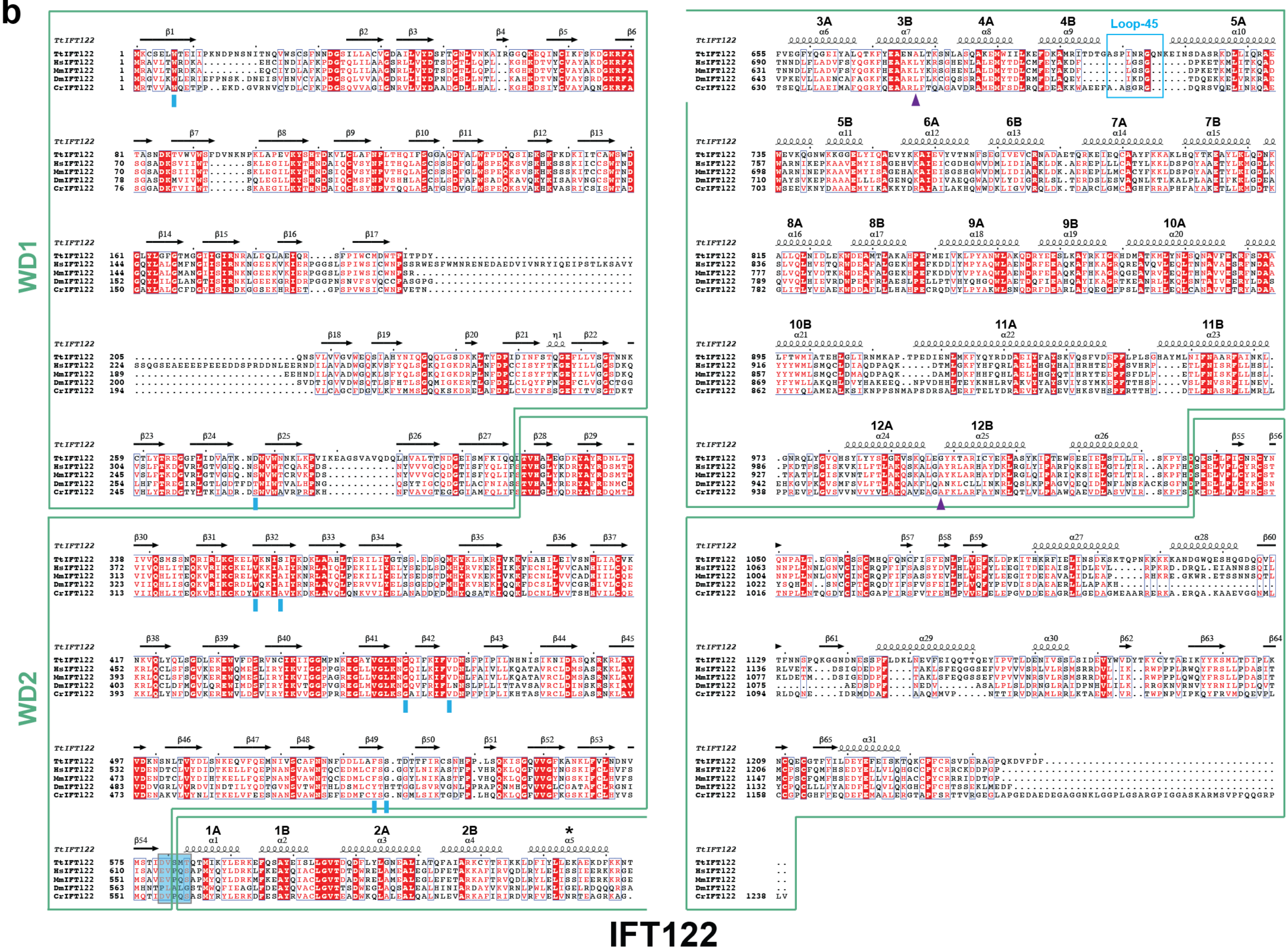


IFT122

**Supplementary Fig. 6**

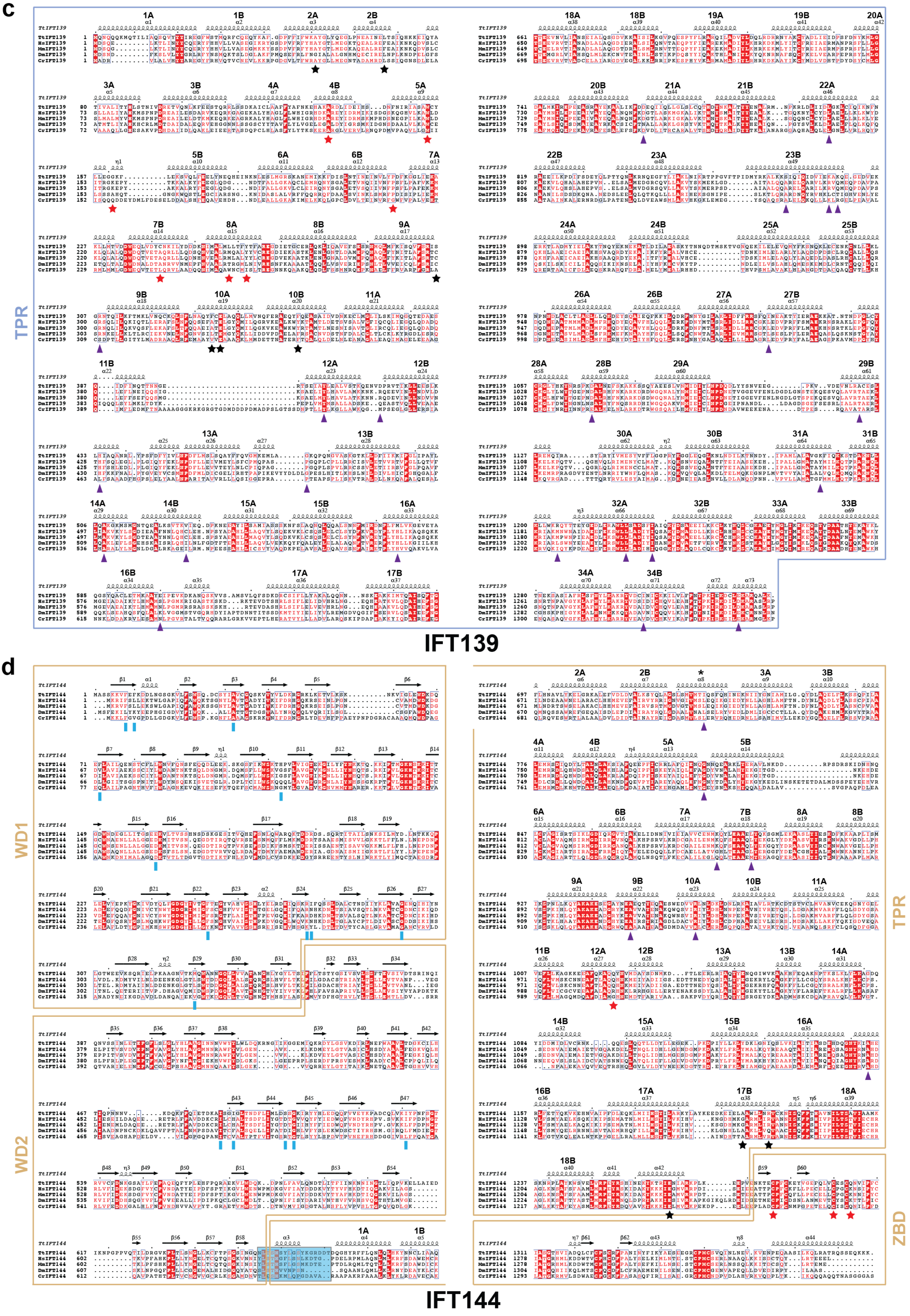

# Supplementary Fig. 6

e

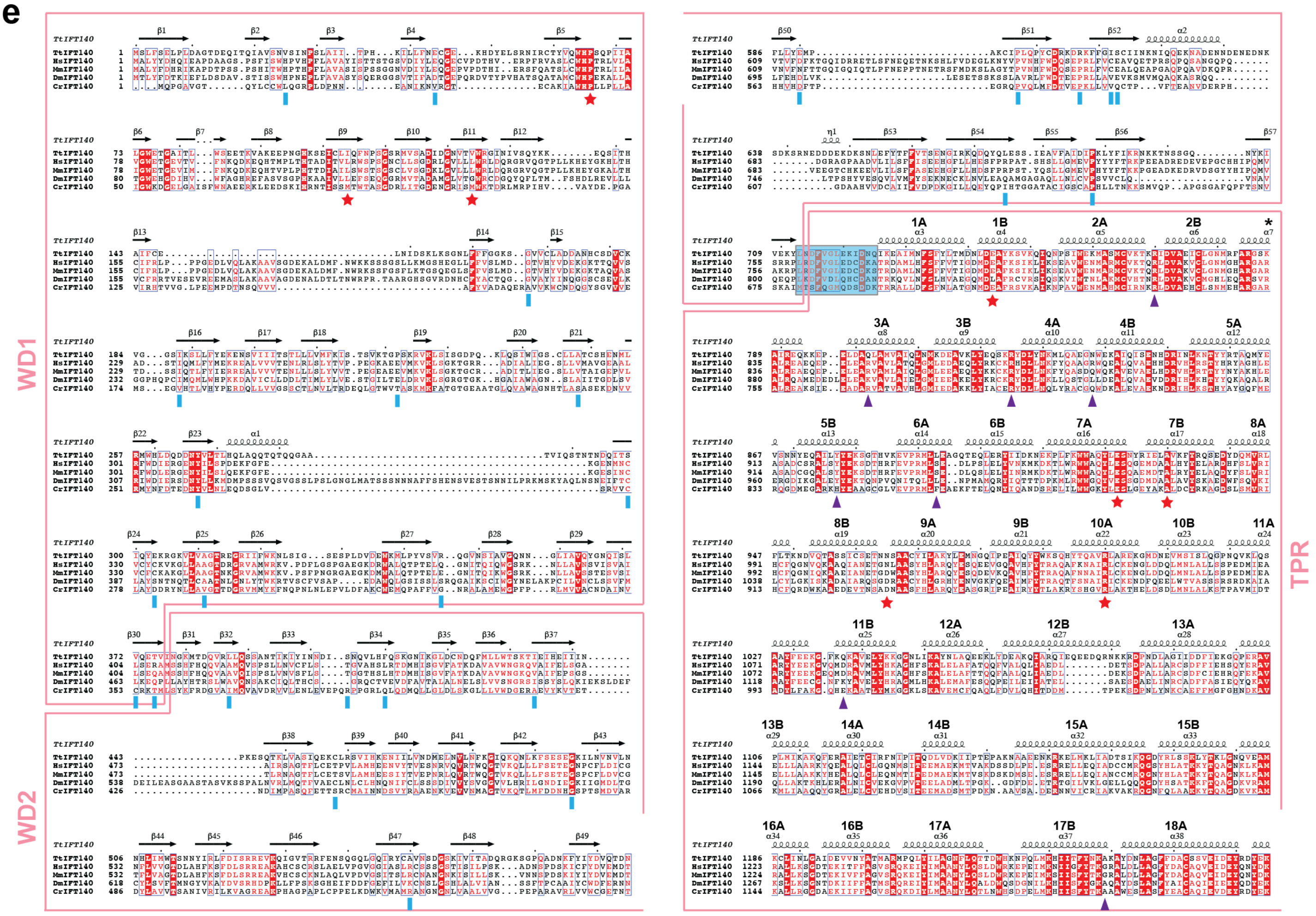


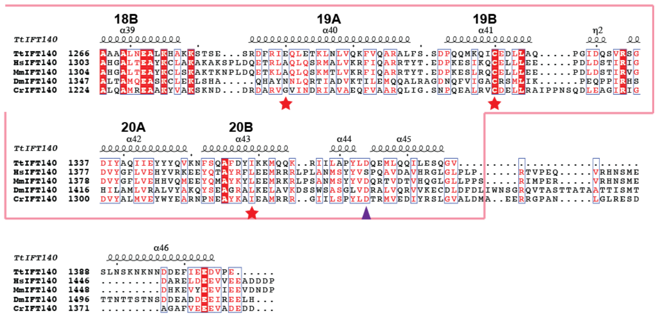

IFT140

f

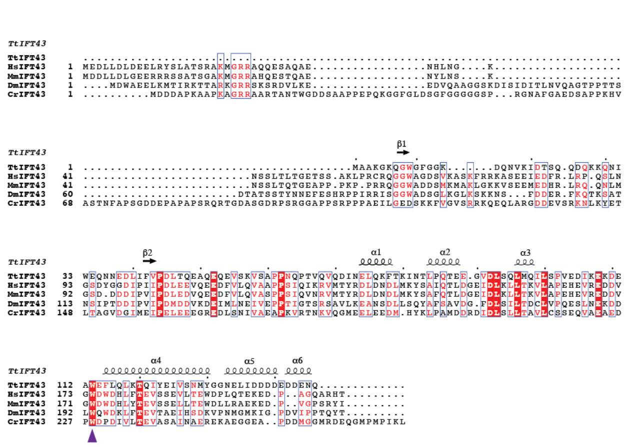

IFT43

**Supplementary Fig. 6 | Multiple sequence alignment of the IFT-A components. a-f,** Sequences of several representative organisms are used for alignment in IFT121 (**a**), IFT122 (**b**), IFT139 (**c**), IFT144 (**d**), IFT140 (**e**) and IFT43 (**f**). The short names Tt, Hs, Mm, Dm and Cr are the abbreviations of *Tetrahymena thermophila*, *Homo sapiens*, *Mus musculus*, *Drosophila melanogaster* and *Chlamydomonas reinhardtii*, respectively. **a,** Accession numbers used for IFT121 are as follows: Q22U89 (Tt), Q9P2L0 (Hs), Q8BND3 (Mm), Q9W097 (Dm) and A8JFR3 (Cr). **b,** Accession numbers used for IFT122 are as follows: Q244W3 (Tt), Q9HBG6 (Hs), Q6NWV3 (Mm), Q9VSJ6 (Dm) and H9CTG6 (Cr). **c,** Accession numbers used for IFT139 are as follows: I7MFN3 (Tt), Q7Z4L5 (Hs), Q0HA38 (Mm), A0A0Q9X6X6 (Dm) and A9XPA6 (Cr). **d,** Accession numbers used for IFT144 are as follows: Q22BP2 (Tt), Q8NEZ3 (Hs), Q3UGF1 (Mm), A1ZBM3 (Dm) and A9XPA7 (Cr). **e,** Accession numbers used for IFT140 are as follows: I7LVZ7 (Tt), Q96RY7 (Hs), E9PY46 (Mm), Q7KTZ4 (Dm) and Q68K27 (Cr). **f,** Accession numbers used for IFT43 are as follows: Q22NF5 (Tt), Q96FT9 (Hs), Q9DA69 (Mm), Q9VK67 (Dm) and A8HYP5 (Cr). The two helices in each TPR motif are distinguished and labeled as helix A and B. Linkers between the WD and TPR domains of IFT122, IFT121, IFT144, IFT140 are highlighted in light blue shadows. The Loop-45 in IFT122 crucial for formation of the elongated and folded IFT-A complexes is highlighted with a cyan rectangle. Mutations that impair cargo binding, interactions of the components within IFT-A, interactions between IFT-As, or interactions between IFT-A and IFT-B are denoted by blue rectangles, purple triangles, red stars and black stars under the sequences, respectively.

**Supplementary Fig. 7**

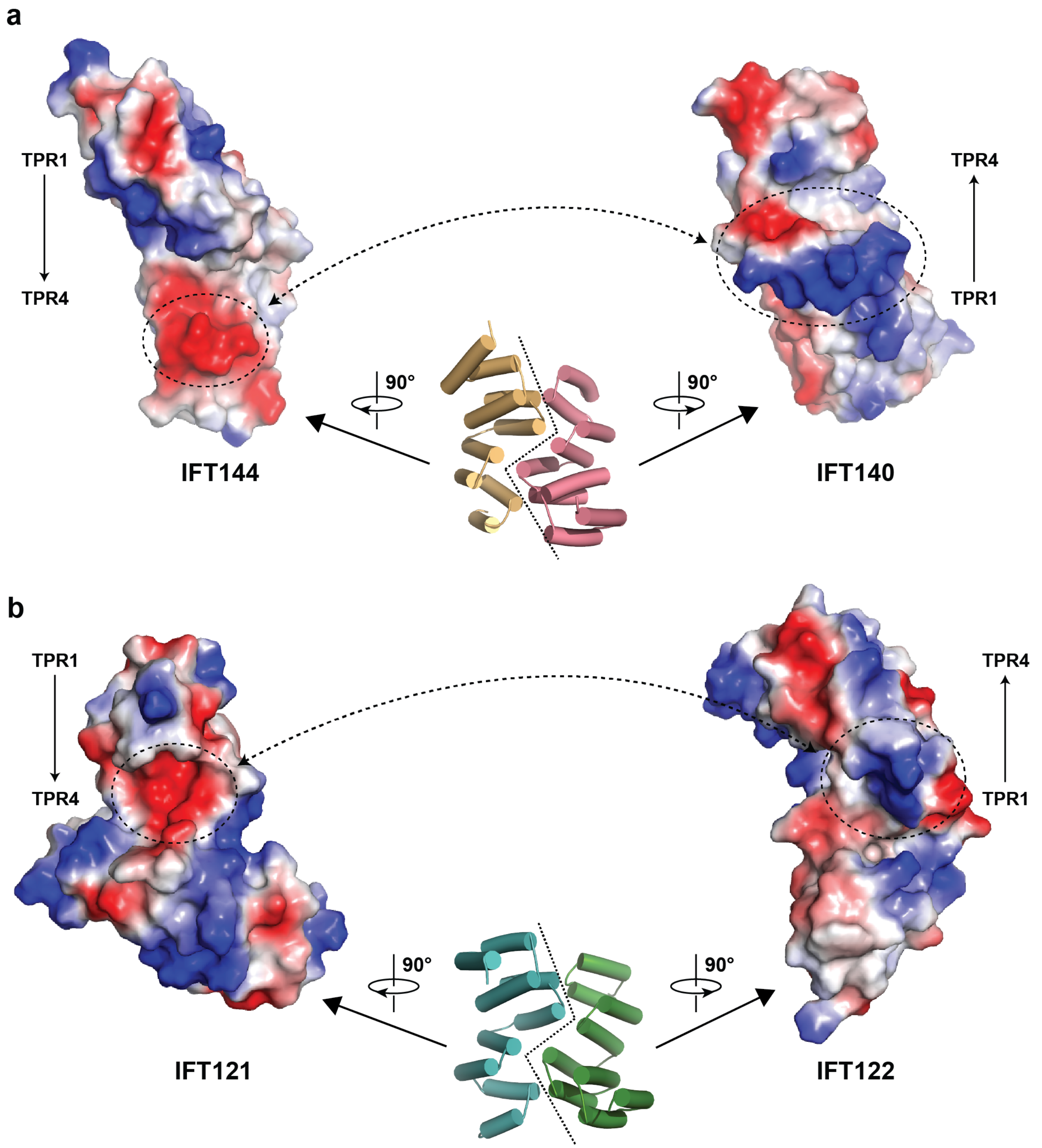

**Supplementary Fig. 7 | Electrostatic surface potential showing the interactions at the TPR junctions.** The opposite electrostatic surfaces important for the IFT144-IFT140 interaction **(a)** and the IFT122-IFT121 interaction **(b)** are highlighted in dashed circles. Positive potential, blue; negative potential, red.

## Supplementary Fig. 8

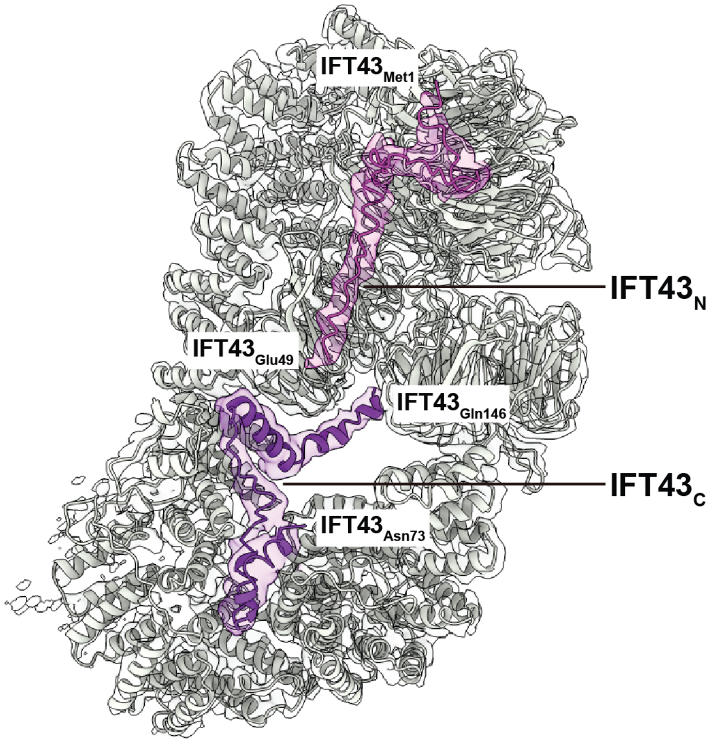

**Supplementary Fig. 8 | IFT43 in the base module.** Atomic models of $IFT43_N$ and $IFT43_C$ are colored in violet and purple respectively, while other IFT-A components and the density map of the base module in grey.

## Supplementary Fig. 9

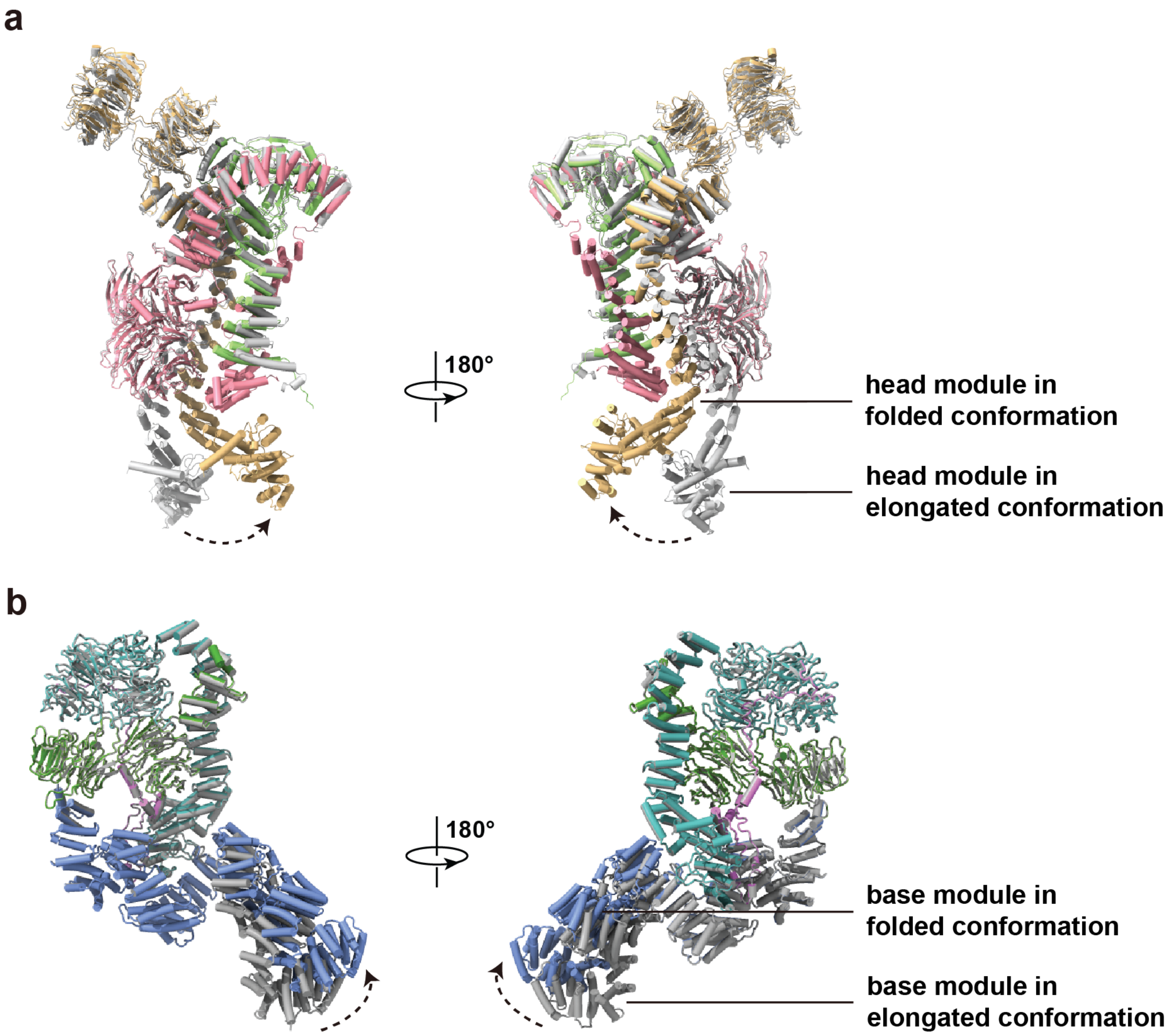

**Supplementary Fig. 9 | Structural alignment of the head and base modules. a-b,** Two opposite views of the head **(a)** and base **(b)** modules. Proteins in the folded state are colored as Fig. 1a, and those in the elongated state in gray. The black arrows indicate the flex of $IFT144_C$ and $IFT139_N$.

## Supplementary Fig. 10

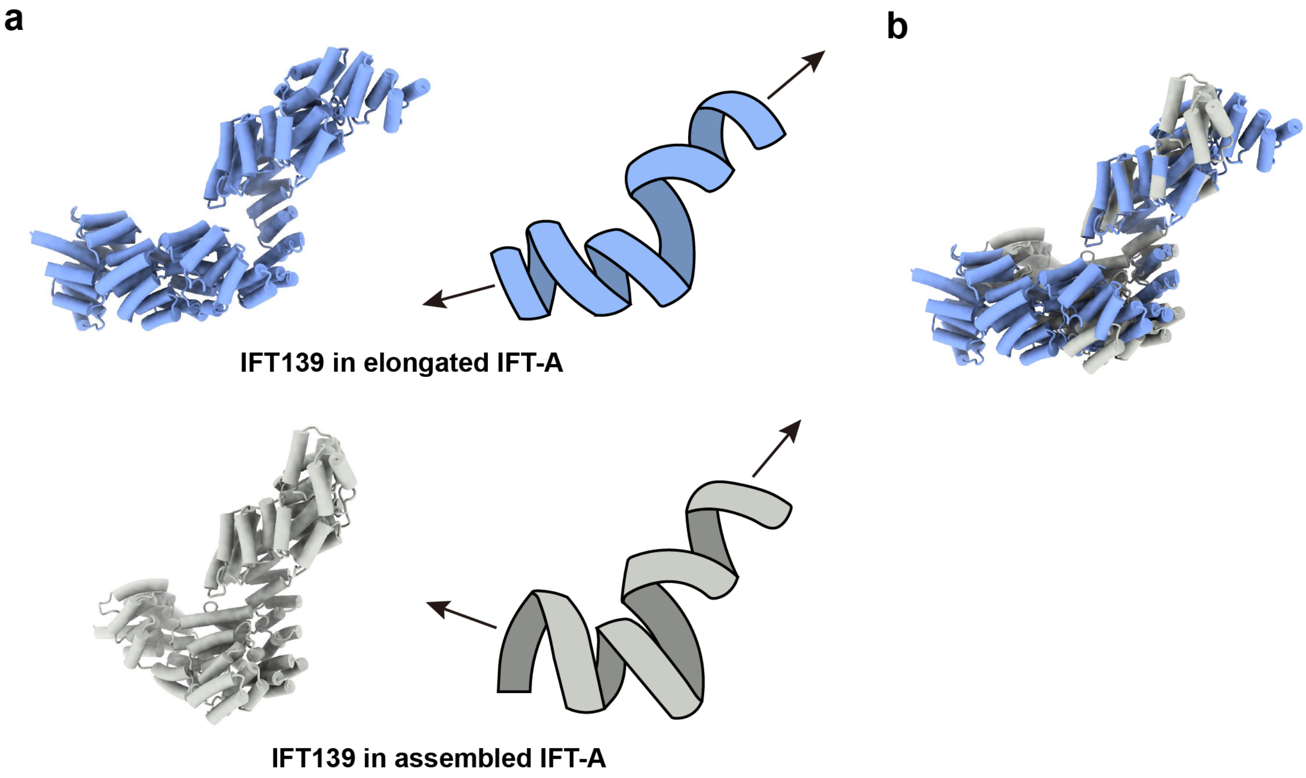

**Supplementary Fig. 10 | Twist in IFT139$_N$ during the incorporation of the elongated IFT-A complex into the anterograde train. a,** Cartoons and schematic diagrams showing the difference between solenoid structures of IFT139$_N$ in elongated state (light blue) and assembled state (gray) of the IFT-A complex. **b,** IFT139$_N$ is superimposed to compare the conformational changes in the two states in **(a)**.

# Supplementary Fig. 11

a

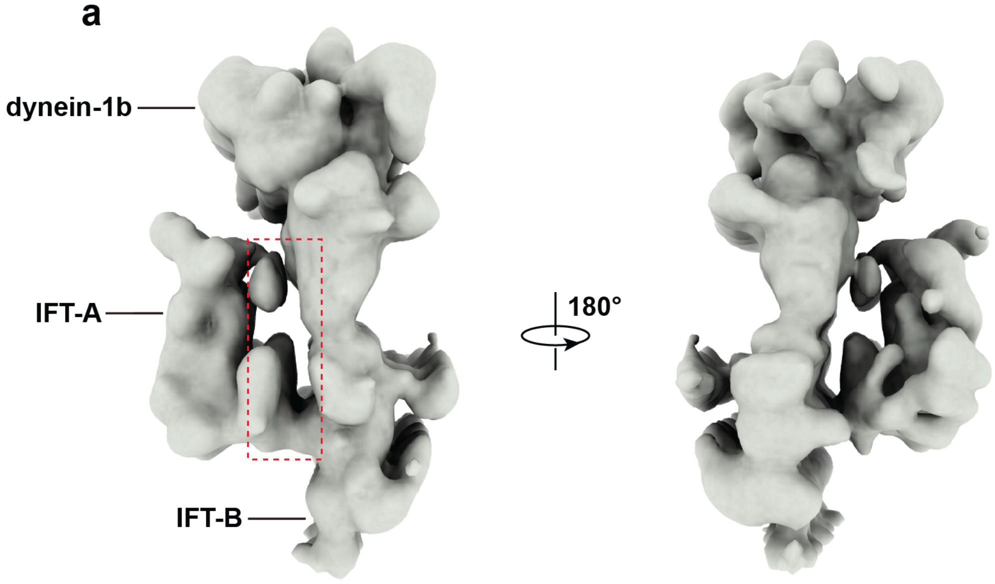


IFT-A/IFT-B train at 29.9 Å
(van den Hoek *et al.*)

b

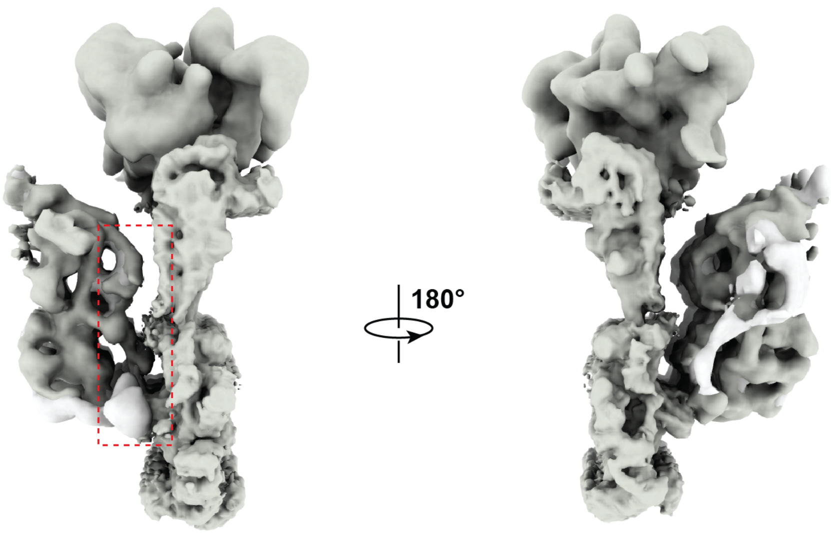


IFT-A (at 20.7 Å) / IFT-B train (at 9.9 Å)
(Lacey *et al.*)

**Supplementary Fig. 11 | Interface between IFT-A and IFT-B in the anterograde train**. **a,** Low-resolution *Chlamydomonas* IFT train map (EMD-15261) at the ciliary transition zone. **b,** Composite map generated by fitting high-resolution structures of IFT-A and IFT-B (EMD-15980 and EMD-15977) into the anterograde IFT train map in (a). Dashed rectangles denote the interfaces between IFT-A and IFT-B.

# Supplementary Fig. 12

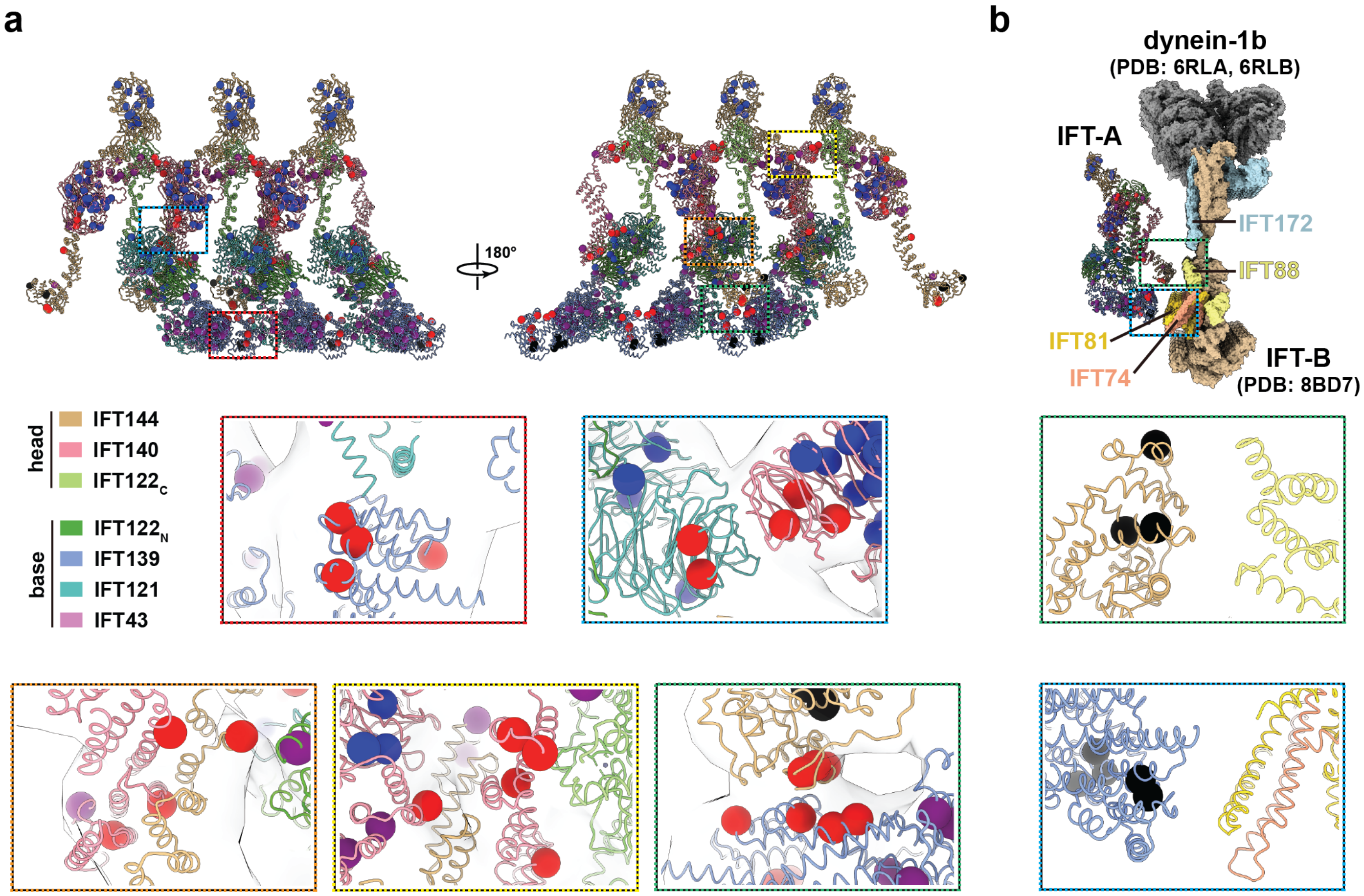

**Supplementary Fig. 12 | Enlarged views of mutations at type-II and -III interfaces.**

**a,** Human disease mutations are mapped onto the assembled IFT-A of anterograde trains. Dashed boxes in colors indicate type-II mutations and closeup views of those mutations at the interfaces are shown below. **b**, The [IFT-A]-[IFT-B] interfaces. Closeup views of the type-III mutations at the [IFT-A]-[IFT-B] interfaces are shown below. Mutations of type I-IV are shown in blue, red, black and purple dots, respectively.
