# Supplementary material for: Structural insight into the intraflagellar transport complex IFT-A and its assembly in the anterograde IFT train
Source: Nat Commun. 2023 Mar 17;14:1506. doi: 10.1038/s41467-023-37208-2 (PMC10023715; doi:10.1038/s41467-023-37208-2)
Supplement: Supplementary file 3 — Description of Additional Supplementary Files [file 41467_2023_37208_MOESM3_ESM.pdf]

File name: Supplementary Data 1

Description: Mass spectrometry data of the purified IFT-A complex.

File name: Supplementary Data 2

Description: Registered pathogenic missense mutations in IFT-A components.

File name: Supplementary Movie 1

Description: The flexibility of the head and base modules in the elongated state with a broader angular distribution.

File name: Supplementary Movie 2

Description: Assembly of IFT-A in the anterograde train.
