# Supplementary material for: Structural insight into the intraflagellar transport complex IFT-A and its assembly in the anterograde IFT train
Source: Nat Commun. 2023 Mar 17;14:1506. doi: 10.1038/s41467-023-37208-2 (PMC10023715; doi:10.1038/s41467-023-37208-2)
Supplement: Supplementary file 8 — Reporting Summary [file 41467_2023_37208_MOESM8_ESM.pdf]

nature portfolio

Corresponding author(s): Ming Lei

Last updated by author(s): Feb 20, 2023

Reporting Summary

Nature Portfolio wishes to improve the reproducibility of the work that we publish. This form provides structure for consistency and transparency in reporting. For further information on Nature Portfolio policies, see our Editorial Policies and the Editorial Policy Checklist.

Statistics

For all statistical analyses, confirm that the following items are present in the figure legend, table legend, main text, or Methods section.

n/a	Confirmed	
☒	☐	The exact sample size (*n*) for each experimental group/condition, given as a discrete number and unit of measurement
☐	☒	A statement on whether measurements were taken from distinct samples or whether the same sample was measured repeatedly
☒	☐	The statistical test(s) used AND whether they are one- or two-sided<br>*Only common tests should be described solely by name; describe more complex techniques in the Methods section.*
☒	☐	A description of all covariates tested
☒	☐	A description of any assumptions or corrections, such as tests of normality and adjustment for multiple comparisons
☒	☐	A full description of the statistical parameters including central tendency (e.g. means) or other basic estimates (e.g. regression coefficient) AND variation (e.g. standard deviation) or associated estimates of uncertainty (e.g. confidence intervals)
☒	☐	For null hypothesis testing, the test statistic (e.g. *F*, *t*, *r*) with confidence intervals, effect sizes, degrees of freedom and *P* value noted<br>*Give P values as exact values whenever suitable.*
☒	☐	For Bayesian analysis, information on the choice of priors and Markov chain Monte Carlo settings
☒	☐	For hierarchical and complex designs, identification of the appropriate level for tests and full reporting of outcomes
☒	☐	Estimates of effect sizes (e.g. Cohen's *d*, Pearson's *r*), indicating how they were calculated

Our web collection on statistics for biologists contains articles on many of the points above.

Software and code

Policy information about availability of computer code

Data collection	EPU-v2.12.1.2782REL
Data analysis	Gctf_v1.06, MotionCor2-v1.0.0, RELION-v3.0.b2, Gautomatch-v0.56, ChimeraX-v1.3, COOT-v0.89, PHENIX-v1.14, PyMOL-v2.0.7, MolProbity-v4.5.2, AlphaFold-2, ResMap-v1.95.

For manuscripts utilizing custom algorithms or software that are central to the research but not yet described in published literature, software must be made available to editors and reviewers. We strongly encourage code deposition in a community repository (e.g. GitHub). See the Nature Portfolio guidelines for submitting code & software for further information.

Data

Policy information about availability of data

All manuscripts must include a data availability statement. This statement should provide the following information, where applicable:

- Accession codes, unique identifiers, or web links for publicly available datasets
- A description of any restrictions on data availability
- For clinical datasets or third party data, please ensure that the statement adheres to our policy

We have provided a full data availability statement in the manuscript.

Human research participants

Policy information about studies involving human research participants and Sex and Gender in Research.

Reporting on sex and gender	N/A
Population characteristics	N/A
Recruitment	N/A
Ethics oversight	N/A

Note that full information on the approval of the study protocol must also be provided in the manuscript.

Field-specific reporting

Please select the one below that is the best fit for your research. If you are not sure, read the appropriate sections before making your selection.

☒ Life sciences ☐ Behavioural & social sciences ☐ Ecological, evolutionary & environmental sciences

For a reference copy of the document with all sections, see nature.com/documents/nr-reporting-summary-flat.pdf

Life sciences study design

All studies must disclose on these points even when the disclosure is negative.

Sample size	Cryo-EM datasets for IFT-A complex were collected three times (23 days in total) , resulting in 538,078, 86,362, 386,801, 199,946, 41,877, 35,628 and 14,020 particles for reconstitution of base module at 3.6 Å, base module at 4.7 Å, head module at 4.2 Å, head module at 4.6 Å, head module at 6.0 Å, IFT-A in elongated state at 8.8 Å and IFT-A in folded state at 8.5 Å, respectively .
Data exclusions	Low-quality and false-positive particles were excluded during 2D and 3D classifications, as a standard image processing practice in cryo-EM.
Replication	Cryo-EM datasets for IFT-A complex were collected three times (23 days in total) in a year. All attempts to replication were successful.
Randomization	N/A. In order to obtain high-quality IFT-A particles and high-resolution IFT-A structures, cryo-EM sample preparation and data processing must be consistent between the three repeats of data colloection.
Blinding	N/A. For structure determination by cryo-EM, millions of IFT-A particles obtained from a set of low-dose cryo-EM images are required for reconstruction of 3D density maps. Then, on the basis of this high-resolution map, atomic models of IFT-A are built according to protein sequence. Blinding is not required.

Reporting for specific materials, systems and methods

We require information from authors about some types of materials, experimental systems and methods used in many studies. Here, indicate whether each material, system or method listed is relevant to your study. If you are not sure if a list item applies to your research, read the appropriate section before selecting a response.

Materials & experimental systems

n/a	Involved in the study	
☒	☐	Antibodies
☐	☒	Eukaryotic cell lines
☒	☐	Palaeontology and archaeology
☒	☐	Animals and other organisms
☒	☐	Clinical data
☒	☐	Dual use research of concern

Methods

n/a	Involved in the study	
☒	☐	ChIP-seq
☒	☐	Flow cytometry
☒	☐	MRI-based neuroimaging

Eukaryotic cell lines

Policy information about cell lines and Sex and Gender in Research

Cell line source(s)	Tetrahymena thermophila strains B2086 and CU428.2 purchased from Tetrahymena Stock Center in Cornell University.

Authentication	None of the cell line used were authenticated.
Mycoplasma contamination	The cell line was not tested for mycoplasma contamination.
Commonly misidentified lines (See ICLAC register)	No commonly misidentified cell lines were used in the study.
